# Antifungal Drugs for Invasive Candida Infections (ICI) in Neonates: Future Perspectives

**DOI:** 10.3389/fped.2019.00375

**Published:** 2019-09-20

**Authors:** Iliana Bersani, Fiammetta Piersigilli, Bianca Maria Goffredo, Alessandra Santisi, Sara Cairoli, Maria Paola Ronchetti, Cinzia Auriti

**Affiliations:** ^1^Neonatal Intensive Care Unit, Department of Neonatology, Bambino Gesù Children's Hospital, Rome, Italy; ^2^Biochemistry Laboratory, Department of Specialist Pediatrics, Bambino Gesù Children's Hospital, Rome, Italy

**Keywords:** neonate, fungal infection, sepsis, lock therapy, polyenes, azoles, echinocandins, candidemia

## Abstract

Fungal infections may complicate the neonatal clinical course, and the spectrum of therapies for their treatment in the perinatal period is limited. Polyenes, Azoles and Echinocandins represent the three classes of antifungal drugs commonly used in the neonatal period. The present review provides an overview about the most recent therapeutic strategies for the treatment of fungal infections in neonates.

## Introduction

### Fungal Infections

Yeasts are commensal organisms which normally colonize mucosal surfaces and skin. However, they display a variety of virulence factors which may potentially allow for the infection of the host organism.

Fungal adhesion to host tissues is of primary importance for tissue colonization. Yeasts exploit either specific (ligand-receptor interactions) or non-specific mechanisms to adhere with different tissue types and inanimate surfaces. The production of the so-called “adhesins,” proteins able to interact specifically with molecules of the extracellular matrix, is essential for fungal adhesion, while the non-specific mechanisms include electrostatic charge, and van der Waals forces (1). In the case of damaged epithelium, higher amounts of extracellular matrix proteins are exposed, thus allowing for an easier fungal adhesion. A further virulence factor is the production of enzymes, such as secreted aspartyl proteases (SAP), phospholipases, lipases, and hydrolytic enzymes, which allow the lysis of the cellular membrane and, therefore, the entrance into the host cells (2). The fungal ability to form intraluminal catheter biofilms also plays a primary role in the colonization of epithelial surfaces and subsequent dissemination. Moreover, *Candida*'s ability to form hyphae is crucial for its virulence and dissemination (3).

Besides fungal virulence factors, a number of host features may facilitate fungal colonization and infection. *Candida* infections develop more easily if the host has impaired defense mechanisms. In preterm neonates, *Candida* represents the third most common causative agent of late-onset sepsis and has a high burden of morbidity and mortality (4, 5). *Candida* infection (ICI) occurs in 4–18% of critically ill neonates, with higher incidence among extremely low birth weight (ELBW) infants (birth weight ≤ 1,000 grams) (4, 6–10). Between 20 and 30% of these infants are likely to die. The mortality rate is comparable between neonates with positive blood cultures and neonates with positive urine cultures (4, 11, 12). Moreover, *Candida* is able to invade virtually all body tissues and possible complications such as blindness, impaired neurodevelopment, and need for surgical corrective procedures may develop after severe ICI in neonates who survive (11, 12). In case of ICI, the central nervous system (CNS) is frequently involved and the higher risk is described in ELBW infants, presenting neurologic involvement in 50–64% of cases (4, 12). When CNS is affected, the mortality rate increases to 30–60% and survivors may develop significant long-term neurological disorders (6–8, 12). In addition to the low birth weight (BW), further risk factors for *Candida* infections include fungal colonization of more than two body sites, the exposure to total parenteral nutrition and to H_2_ receptor antagonists, to antenatal and postnatal antibiotics, and to corticosteroids (4, 11). Among invasive procedures, the presence of indwelling catheters, the exposure to mechanical ventilation and to abdominal surgery are well-known risk factors in the preterm infants (11).

Fungal burden varies between different countries and hospitals (4, 13–16), and much of this variability is explained by the variability of procedures, drugs, and feeding practices used in different clinical contexts (10).

At least 15 different species of pathogen *Candida* are detectable in humans, although >90% of ICI are caused by the five most frequent pathogen species, i.e., *Candida albicans, Candida parapsilosis, Candida glabrata, Candida tropicalis*, and *Candida krusei*. *Candida albicans* and *Candida parapsilosis* are more frequently associated with disease in neonates (17, 18). Each *Candida* species may vary in terms of virulence properties (19–24). Possible virulence factors include the ability to undergo phenotypic switching, the expression of adhesion molecules on cell surface allowing a higher attachment to host structures, and the production of hydrolytic enzymes (19). Some fungal strains also show a high propensity to form biofilms on the surface of devices, such as central line catheters, making these strains particularly difficult to treat (25).

The diagnosis of ICI is challenging. Signs and symptoms of IC may be non-specific and are often subtle, therefore a combination of clinical, radiological, and mycological assessments is required. Besides culture isolation (blood, urine, cerebrospinal fluid, peritoneal fluid, tracheal aspirate), other microbiologic techniques to diagnose an ICI include direct microscopic examination, histologic examination of the involved tissues, assessment of fungal antibodies, and of fungal antigens (galactomannan, 1,3-β-D-glucan) by enzyme-linked immunosorbent assay (ELISA) test or immunofluorescence and molecular diagnosis by real-time Polymerase Chain Reaction determination of fungal DNA. Although fungal strains usually easily grow in culture medium, their identification requires large volumes of blood, which are difficult to collect in the preterm neonate. This may explain why, in this special population of patients, blood cultures may be negative for a large number of fungal bloodstream infections. Furthermore, 50% of fungal sepsis with negative blood cultures show a positive culture of the cerebrospinal fluid, underlying the complexity of an ICI diagnosis (12).

## New Options For Antifungal Therapy in the Neonatal Age

The cell membrane and cell wall of fungi consist of a phospholipid bilayer, including ergosterol, chitin and chitosan, beta 1,3 and beta 1,6 glucans, mannoproteins, and other components, in various combinations.

Antifungal drugs act by means of different mechanisms, such as interference with cell membrane synthesis, with cell wall synthesis and stability, and with fungal DNA/RNA synthesis (26).

The target of Polyenes and Azoles is ergosterol, the predominant sterol in many pathogenic fungi. Echinocandins block cell wall synthesis by inhibiting the enzyme 1,3 beta glucan synthase, antimetabolites inhibit the protein syntesis.

Safe and effective therapeutic strategies for the treatment of ICI in the neonatal period are limited (10, 27, 28), and Polyenes, Azoles and Echinocandins represent the three classes of antifungal drugs more commonly used in infants [Table 1; (34)].

**Table 1:** Antifungal drugs currently used in infants with ICI.

**Class and mechanism**	**Drug**	**Indication**	**Recommended neonatal dose**	**Side effects**
**Polyenes**Bind to ergosterol in the fungal cell membrane. Depolarize the membrane. Cause formation of pores that increase permeability to proteins and electrolytes. Cause the cell death.	Amphotericin B deoxycholate (D-AMP-B)	Therapy of systemic and deep fungal infections Empirical therapy of invasive fungal infection	0.5–1.5 mg/kg/day	Nephrotoxicity: drop in renal blood flow and glomerular filtration rate; increase in serum creatinine and increase in blood urea nitrogen; wasting of Na+, K+, and Mg++; impaired urinary acidification and concentration; renal tubular acidosis.
	Amphotericin B lipid complex (ABCD)		3–5 mg/Kg/day	All three lipidic formulations reduce, but do not eliminate, nephrotoxicity due (presumably) to their reduced distribution of drug to the kidney.
	Amphotericin B lipid complex (ABLC)		5 mg/kg/day	
	Liposomal Amphotericin B (L-AMP-B)		1–5 mg/kg/day	
**Azoles**Fungistatic drugs. Inhibit the fungal cytochrome P-450 3-A dependent enzyme 14-alpha demethylase, interrupting the synthesis of ergosterol, leading to the depletion of ergosterol in the cell membrane and inhibition of fungal growth.	Fluconazole (for therapy)	Therapy of systemic and deep fungal infections in neonates not previously treated with this drug. Empirical therapy of invasive fungal infection in neonates not previously treated with this drug.	12 mg/kg/day	Transient elevation of plasma levels of creatinine or hepatic AST and ALT (> 3-folds).
	Fluconazole (for prophylaxis)	Prophylaxis of invasive candidiasis in ELBW and VLBW neonates	3–6 mg/Kg/day 2 or 3 times in a week	Transient elevation of plasma levels of creatinine or hepatic AST and ALT (> 3-folds) May cause prolonged QT syndrome if used together with drugs prolonging the QT interval.
**Echinocandins**Block fungal cell wall synthesis by inhibiting the enzyme 1,3-beta glucan synthase.	Caspofungin^*^	Therapy of systemic and deep fungal infections Empirical therapy of invasive fungal infection	25 mg/m^2^/day 0.5–2 mg/Kg/day	Hypokalemia, thrombophlebitis, transient elevation of hepatic AST and ALT
Depletion of glucan polymers in the fungal cell wall, unable to withstand osmotic stress.	Anidulafungin^**^	Therapy of systemic and deep fungal infections Empirical therapy of invasive fungal infection	1.5 mg/kg/day	Transient elevation of plasma levels of hepatic AST, ALT, and bilirubin (<3-folds).
	Micafungin	Therapy of systemic and deep fungal infections Empirical therapy of invasive fungal infection	4–15 mg/kg/day	Transient elevation of plasma levels of hepatic AST, ALT, and Gamma GT.

* The use of body surface area as a metric of size to establish Caspofungin dose may be inaccurate in neonates.

** *Limited neonatal pharmacokinetic data are available. Inconclusive data about the dosing of Anidulafungin sufficient to penetrate brain tissue. To be generally avoided in neonates. See Kliegman et al. (29), Auriti et al. (30), Natarajan et al. (31), Hope et al. (32), Cohen-Wolkowiez et al. (33)*.

Conventional Amphotericin B deoxycholate (D-AMPH-B), and fluconazole, represented the only therapeutic choice for candidiasis in neonates and infants for many years. In recent years, due to the resistance of some *Candida* spp. against fluconazole (although with difference rates worldwide), the use of echinocandins has progressively increased, thanks also to the more specific mechanism of action, which limits the side effects of the therapy. Nevertheless, most of the available data refer to infants (35), and only a minority of them refer specifically to the neonatal period. Furthermore, the newer therapies are sometimes more expensive than the traditional ones.

**Figure 1:**
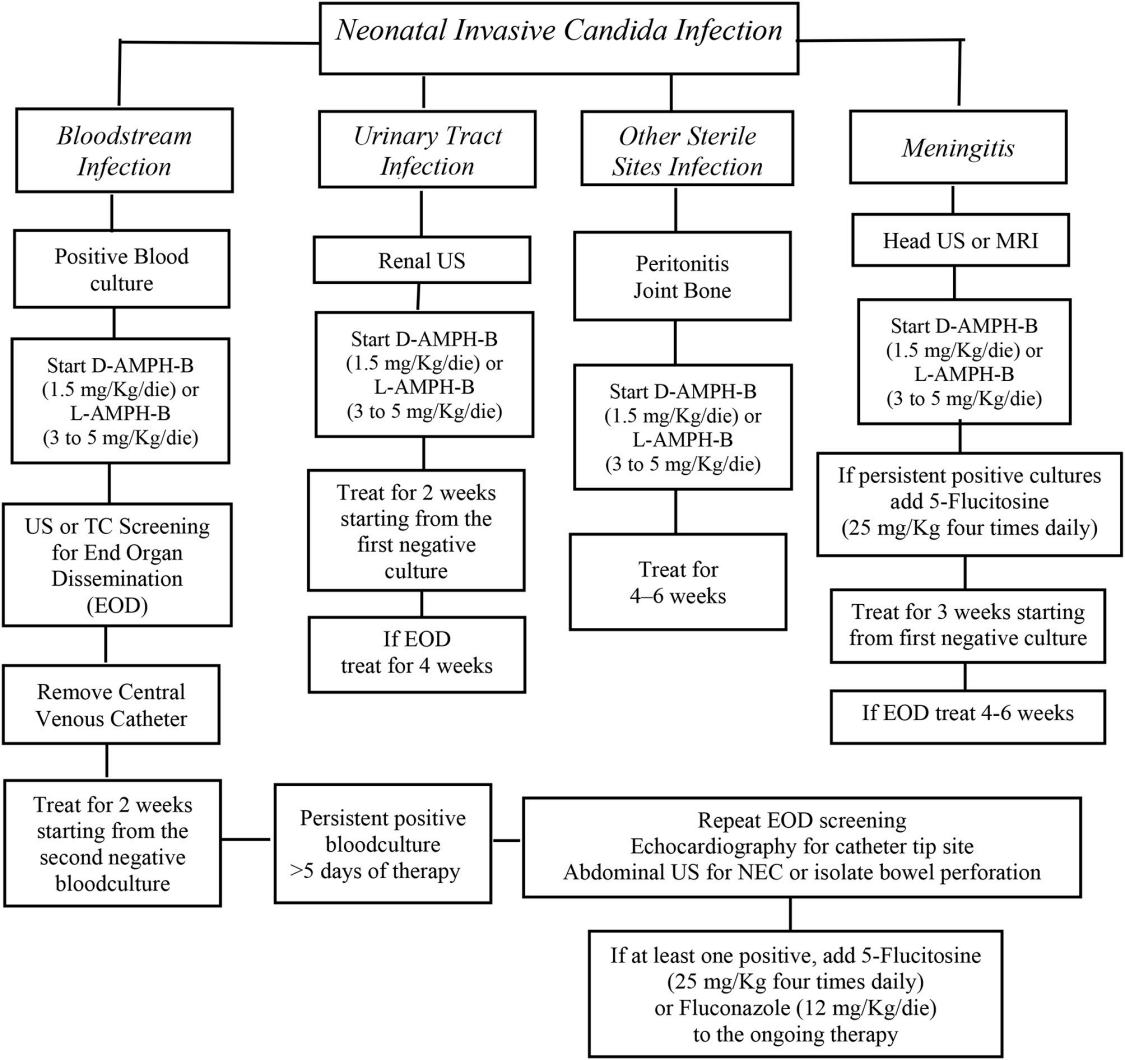
AmB deoxycholate should be started at 1 mg/kg intravenous daily and can be increased up to 1.5 mg/Kg/die. An alternative option is liposomal AmB that should be started at 3–5 mg/kg daily. The addition of 5-flucytosine 25 mg/kg four times daily at should be considered as salvage therapy in patients not responsive to initial AmB therapy or with End Organ Dissemination (EOD). Fluconazole, 12 mg/kg daily, is recommended for Candida strains that are susceptible to fluconazole, in babies who had not a previous fluconazole prophylaxis. Algorithm for the initial treatment of Invasive Candida Infections in neonates [from (28), modified].

The administration of the most appropriate drug and at the optimal dosing is obviously crucial (Figure 1) (28). However, for most of the antifungal drugs, the appropriate dosages in neonates are still discussed [Table 1; (26, 29–33)].

### Polyenes

Conventional amphotericin B deoxycholate (D-AMPH-B) has represented the standard of care for the IC therapy since 1950, when it was introduced in the current clinical management of fungal infections, in adults, and children. D-AMPH-B is a polyene macrolide antifungal drug isolated from *Streptomyces nodosus* (34, 36) By its interaction with ergosterol, residing on the fungal cell wall, D-AMPH-B depolarizes the cell membrane and causes the formation of pores, which increase the permeability to proteins and electrolytes, leading to cell death (34). The efficacy correlates with the dose, following the maximum concentration (Cmax)/Minimal Inhibiting Concentration (MIC) ratio and ranging from 4 to 10. D-AMPH-B has fungicidal effects against a spectrum of fungi such *Candida* species, *Aspergillus* species, *Zygomycetes*, and dimorphic fungi (37). In addition, its action is enhanced by the release of reactive oxygen species (ROS) (36). Sporadic resistance against D-AMPH-B has been reported for some fungal strains, but clinically relevant resistance is uncommon (37).

Since D-AMPH-B is poorly absorbed through the gastrointestinal tract, it is administered intravenously. D-AMPH-B decreases renal blood flow and glomerular filtration rate, thus it has a potential nephrotoxicity that limits the total dose that can be given to neonates (38). As D-AMPH-B is mostly protein-bound, its penetration into the extracellular spaces, such as the cerebrospinal fluid, is poor (34).

To decrease the incidence of adverse effects, including hypokalemia or nephrotoxicity described after long lasting D-AMPH B administration, Amphotericin B was combined with lipids and a new antifungal drug was developed. Three formulations of lipid amphotericin B are currently available: (1) amphotericin B lipid complex-ABLC, complexed with dimyristoyl-phosphatidylcholine, and phosphatidylglycerol, whose configuration is ribbon-like; (2) amphotericin B colloidal dispersion (ABCD), complexed with cholesteryl sulfate, that has a disk-like structure; (3) liposomal amphotericin B (L-AMPH-B), complexed with hydrogenated soy phosphatidylcholine, distearil-phosphtidyl-glycerol, and cholesterol. Unlike the other lipid formulations, L-AMPH-B is a true liposome composed of mono-lamellar lipid vesicles, allowing the drug to reach targeted tissue. To date, L-AMPH-B is currently used also in infants and children (39). Its antifungal activity is effective against many clinically aggressive agents, including *Candida* spp., *Aspergillus* spp., and filamentous molds such as *Zygomycetes* (39, 40). The newer lipid formulations release L-AMPH-B exactly into its action site on the fungal cell membrane. Furthermore, a lower toxicity was detected in mammalian cells, thanks to the higher stability of the lipid formulation, allowing higher dosages of L-AMPH-B to be administered (34), regardless of birth weight, gestational age or chronological age of the newborn (41).

Due to the association of L-AMPH-B with liposomes, the risk for nephrotoxicity and infusion-related toxicity is lower compared to conventional amphotericin B (40). Moreover, it has higher tissue concentrations, with the highest levels detected in the liver, spleen, kidneys, and lungs (42).

The recommended dose for L-AMPH-B is 3–5 mg/Kg/day, whereas for D-AMPH-B the dose starts from 0.5 to 0.7 mg/Kg/die to 1.5 mg/Kg/day (4, 17, 43). A recent study demonstrated that a high dose of L-AMPH-B is effective and well-tolerated in very low birth weight (VLBW) neonates affected by candidiasis (44). Although L-AMPH-B is the most widely used antifungal agent in infants and D-AMPH-B the most used in newborns, there are no available prospective randomized trials in neonates, providing information about the pharmacokinetic properties of these drugs and their safety.

### Azoles

The second major class of antifungal agents available for the treatment of preterm infants with invasive fungal infection is the azole group. This includes triazoles (fluconazole voriconazole and imidazoles for topical use (miconazole ketoconazole). Triazoles, in particular fluconazole, are used more commonly in neonatal practice, and appear to be a safe treatment for newborn infants (34). Agents of the triazole class, including the recently issued isavuconazole®, exert antifungal activity through the inhibition of sterol 14-a-demethylase (Erg11p). This enzyme of the cytochrome P450 family is responsible for a key demethylation step in the ergosterol biosynthetic pathway. Ergosterol is typically the predominant sterol found in the membranes of fungi, including *Aspergillus, Candida*, and *Mucorales*. It is responsible for the regulation of membrane integrity, fluidity, and permeability. Inhibition of Erg11p blocks the production of ergosterol, diverting ergosterol precursors toward alternative biosynthetic pathways. A portion of these averted intermediates converts to toxic 14-a-methylsterols, which pack more loosely into lipid bilayers leading to leaky and unstable membranes (45). All of the azole antifungals inhibit cytochrome P450 enzymes to some degree. Thus, clinicians must carefully consider the influence on a patient's drug regimen, when adding or removing an azole. Common polymorphisms in the gene encoding the primary metabolic enzyme for voriconazole result in wide variability of serum levels. Drug–drug interactions are common with voriconazole and should be considered when initiating and discontinuing treatment with this compound (27). The most frequently reported side effect is a transient elevation of plasma levels of creatinine or hepatic enzymes, described in about 5% of infants treated with fluconazole (34). Fluconazole is readily absorbed, with oral bioavailability resulting in concentrations equal to ~90% of those achieved by intravenous administration. Absorption is not affected by food consumption, gastric pH, or disease state. Among the triazoles, fluconazole has the greatest penetration into the cerebrospinal fluid (CSF) and vitreous, achieving concentrations of >70% of those in serum. This is the reason why it is often used in the treatment of CNS and intraocular *Candida* infections (27). The efficacy correlates with the dose/24 h, following the Area Under the Curve (AUC)/MIC ratio, that should be higher or equal to 25 for a successful therapy. In neonates, the dosage is 12 mg/Kg/die for 3 weeks (46, 47) regardless of birth weight or gestational age. Blood level measurements might be of help to monitor fluconazole concentrations during the treatment period. In term neonates, fluconazole plasma half-life is ~70 h (30 h in adults) whereas in preterms it is 73 h at birth, 53 h at 6 days of age, and 46 h at 12 days of age. These pharmacokinetic characteristics make fluconazole an attractive candidate for the prevention of ICI, mainly in premature infants, allowing for infrequent administration (48). Moreover, fluconazole is minimally (12%) bound to plasma proteins, penetrates CSF, and achieves saliva and lung concentrations that are 1.3 and 1.2 times the plasma levels, respectively, thereby providing higher concentrations at key areas of colonization (32, 48–53).

Voriconazole is available in oral and intravenous formulations and is the primary therapy for invasive aspergillosis. Consistent with voriconazole time-dependent effect, Cmin >1–2 mg/L is a good predictor of successful clinical outcome in both adults and children. Voriconazole side effects include visual disturbances, elevated hepatic transaminases, and skin photosensitization (13–30%). In adults, concentrations above 4–5.5 mg/L correlate with toxicity (39). Voriconazole must be taken before or after a meal. In children (2–12 years), voriconazole is administered intravenously at 9 mg/kg once every 12 h for the 1st day, then at 8 mg/kg once every 12 h. Orally it should be administered at 9 mg/kg twice in a day (max: 350 mg each dose) for ages ranging 2–14 years (32). However, voriconazole is not recommended in children <2 years and infants.

Isavuconazole is a recently approved expanded-spectrum triazole with excellent *in vitro* activity against *Candida* species. Available in both oral and cyclodextrin-free intravenous formulations, isavuconazole has a broad spectrum of activity including yeast, dimorphic fungi, and various molds, as well as a favorable adverse effect profile and less substantial drug-drug interactions than other triazoles. Isavuconazole is currently indicated for the treatment of invasive aspergillosis and invasive mucormycosis, and the agent is currently being investigated for an indication in the treatment of candidemia and ICI (39). Preliminary analysis of the recently completed large international double-blind trial comparing isavuconazole to an echinocandin for ICI suggests that isavuconazole does not meet criteria for non-inferiority (personal communication, Astellas US) (50). Although much of the role of isavuconazole remains to be revealed by phase IV experience, a broad spectrum of activity, minimal safety concerns, and proven efficacy in the treatment of invasive mold infections, certainly make this latter triazole a welcome addition to the antifungal armamentarium (39).

### Fluconazole Prophylaxis

*Candida* species colonize the skin and mucous membranes of about 77.1% of preterm infants within 4 weeks of admission to the NICU and can progress to fungal invasive infection (51). The colonization of more than one body site by *Candida* is one of the well-known risk factors for ICI in preterm neonates. Then, critically ill neonates benefit greatly from antifungals administered for prophylaxis, which seems to limit fungal colonization and the progression of systemic infections. The use of fluconazole as antifungal prophylaxis is supported by robust studies showing the efficacy and safety of this drug (52–56). Taken together, all data suggest that prophylactic administration of fluconazole, 3–6 mg/kg/dose twice weekly, is appropriate for all neonates and results in a reduction in Candida colonization and a 91% decrease of ICI. Since 2001, Kaufmann and colleagues have demonstrated the efficacy of intermittent administration of fluconazole at a low dose in the prevention of ICI in high risk infants. (53). In a prospective, randomized, clinical trial, Kaufmann evaluated the efficacy of fluconazole prophylaxis vs. placebo in 100 preterm infants. Fungal infection developed in 20% of the infants in the placebo group and in none of those in the fluconazole group. These results have been confirmed in 2007 by an elegant study by Paolo Manzoni. The authors showed that fluconazole administered as prophylaxis twice in a week to very low birth weight infants reduced the incidence of ICI by up to 4% compared to 13.2% in the placebo group (55). Although in this last study the effect on the Candida colonization was not clear, the decrease of ICI was surprising. Fluconazole's long half-life in neonates allows an intermittent administration for the prophylactic treatment. In neonates the mean serum peak concentration of fluconazole increases during the first week but decreases during the second week of life (48). Therefore, fluconazole prophylactic dosage varies according to the chronological age. The dose scheme is 3–6 mg/Kg/dose once a day, 2 times a week in the first 2 weeks of life whereas, from the third week of life, prophylaxis should be administered every day. The benefit of prophylaxis may be less evident in care settings where the incidence of ICI is <2%. In this context the recommendation is to decide to start prophylaxis case by case, in relation to the presence of risk factors for ICI. When the incidence of infections is at least 5% it is advisable to administer fluconazole prophylaxis as a routine treatment to babies of extremely low birth weight or to those of higher birth weight with specific risk factors (32).

### Echinocandins

The possible occurrence of adverse reactions in neonates and the development of fungi resistance to conventional antifungal drugs led to the exploration of new molecules as alternative therapies against systemic fungal infections (56). Although partially studied in neonates, Echinocandins are more and more frequently used in the treatment of disseminated candidiasis in such delicate patients. Echinocandins are semisynthetic cyclic lipopeptides, that block the synthesis of the fungal cell wall, by inhibiting the enzyme (1 → 3)-β-D-glucan synthase complex. The result is a glucan-depleted cell wall susceptible to osmotic lysis (57–60). This target is unique to fungi, as the 1,3-β-D-glucan is not present in mammalian cells, thus contributing to the favorable toxicity profile of the drug and minimal adverse effects (61). The enzyme complex (1 → 3)-β-D-glucan synthetase, contained within the fungal cell wall, is composed of the catalytic subunits FKS1p, FKS2p, and the Rho1p protein. FKS1p is the major subunit, which determines the remodeling of the cell wall in fungi. Mutations in the genes FKS1 and FKS2, encoding proteins, are responsible for fungal drug-resistance. Rho1p is a protein that regulates or stops the synthesis of (1 → 3) -β-D-glucan (61). The proportion of glucan in the fungal cell wall varies widely between fungal species and is predictive of fungicidal activity of echinocandins against *Candida* spp., including fluconazole-resistant spp. Echinocandins have a fungistatic action on the *Aspergillus* spp. and are not active against *Candida* neoformans, *Zygomycetes*, and dimorphic fungi (61, 62).

The three Echinocandins currently available are caspofungin, anidulafungin, and micafungin, and are only available as parenteral preparations. All of them have an optimal spectrum activity against various *Candida* spp. (61–63). Caspofungin represents an appropriate alternative for the therapy of systemic candidiasis in preterm neonates when there is lack of response, resistance or toxicity to other antifungal agents such as D-AMPH-B, or fluconazole (64). Its action is fungicidal against *Candida* spp. and many fungal strains resistant to AMPH-B and triazoles. Plasma concentrations in neonates are slightly more elevated compared to older pediatric patients and adults, and is without any observed adverse safety outcomes. At 25 mg/m^2^ daily, caspofungin is generally well-tolerated in neonates (65) but pharmacokinetics, safety, and efficacy data are lacking until now (31, 64, 65). Anidulafungin is currently not registered and authorized for pediatric use, although pharmacokinetic studies in pediatric and neonatal populations have already been conducted and this Echinocandin may be an option in the future.

Among the Echinocandins, micafungin is the most studied Echinocandin in neonates and the only approved by both European Medicine Agency (EMA) and United States Food and Drug Administration (US-FDA) for younger children (66–71). The few data available in the literature on neonatal pharmacokinetics of micafungin limit the possibility of recommending it as a first-choice drug in the therapy of systemic candidiasis. Moreover, recommendations for the optimal dosage of micafungin in neonates are still not clarified, though, the U.S. FDA approved a dosage of 2 mg/kg/day in infants 4 months of age or older (72). Similar dosing is recommended by the EMA-approved label, although a warning about the potential hepatotoxicity of micafungin has been issued (73). Nevertheless, according to preclinical models and bridging studies about CNS-related candidiasis (74, 75), in neonates the administration of doses higher than 2 mg/kg/day seems to be necessary. Babies younger than 4 months and neonates with VLBW (<1,500 g) seem to require doses from 7 to 15 mg/kg/day to achieve therapeutic plasma levels, compared to 1–4 mg/kg/day, which is the optimal dosage in older children and adults. Although micafungin levels in CSFare relatively low, the daily administration of 8–10 mg/kg/day of micafungin seems suitable, according to pharmacokinetics/pharmacodynamic models in neonates and very preterm neonates, also in the presence of *Candida* localizations in the CNS (76). The clearance of micafungin seems greater in neonates than in adults, due to an unclear mechanism (67). The low level of plasma proteins, characteristic of the preterm infant, could be one of the reasons for the increased micafungin clearance. The micafungin molecule has a high molecular weight and is highly bound to plasma proteins. This binding seems about 8 times lower in neonates than in adults, with an increase in the proportion of free drug, that can be quickly eliminated (67).

Our group analyzed 18 preterm neonates and infants with ICI, three with Candida meningitis, who received, for at least 14 days, 8 to 15 mg/kg/day of intravenous micafungin. Overall, 78.2% of neonates had clinical resolution of their infection. High doses of micafungin were well-tolerated and showed pharmacokinetic profiles predictive of a positive effect. A significant increase in alkaline phosphatase levels was observed. Increased gamma-glutamyl-transferase (GGT) levels were also recorded in three patients treated with 10- to 15- mg/kg/day of micafungin, and improvement of the GGT level was achieved after dose reduction (30).

Clinical efficacy and safety data with newly proposed dosing in neonates needs further evaluation (30), since major discrepancies exist concerning the optimal dosage of micafungin (10, 66–68, 77) for the treatment of systemic candidiasis in the neonatal age. Micafungin is metabolized mainly in the liver, by arylsulfatase, catechol-O-methyltransferase, and by isoenzymes (3A4, 1A2, 2B6, and 2C) belonging to cytochrome P450 system and interactions with other drugs are few (66, 68, 69). The major route of elimination is non-enzymatic degradation (27). The excretion is mainly via feces. Differences in the hepatic metabolism of micafungin between infants and adults have not been demonstrated. Drug levels in urine are relatively low.

The most frequent adverse events include neutropenia, jaw and joint pain, rash, increased hepatic enzymes, abnormal Liver Function Tests, and two serious adverse events: hyperbilirubinemia and increased serum creatinine (66). Due to its hepatic metabolism (68), micafungin use should be avoided in cases of liver diseases, whereas it should represent the first-line choice in case of renal impairment. Micafungin displays concentration-dependent fungicidal killing, and in animal models, efficacy correlates best with AUC:MIC ratios (78). Analysis of adult clinical data for the treatment of systemic candidiasis found that an AUC:MIC ratio over 3,000 predicts better mycological response (79).

Considering the ability of *Candida* strains to develop catheters intraluminal biofilms, micafungin has also been used to perform the catheter lock therapy (72, 73). Compared to amphotericin B or fluconazole, micafungin seems to be preferable for this procedure, thanks to its ability to penetrate the biofilm (63).

### Antifungal Lock Therapy of Central Venous Catheters (CVC)

When a systemic candidiasis is diagnosed, prompt catheter removal is recommended both in adults and in children (80). In neonates, a delayed CVC removal when fungal infections occur seems to be associated with significantly increased mortality (80). A delay in CVC removal (more than 1 day after initiation of systemic antifungal treatment) was also associated with impaired neurodevelopment compared with infants in which CVC was promptly removed at the onset of infection (4). Moreover, the timing of catheter removal also affects candidemia duration. Benjamin et al. showed that the time to clear *Candida* from the blood was equal to 5 days in neonates in whom the CVC was removed immediately after diagnosis of candidemia vs. 7.3 days in those with late removal (4). However, catheter removal may be problematic in very ill neonates requiring CVCs for long-term parenteral nutrition and/or life-saving therapies, and CVC reinsertion may be challenging. Therefore, the need for CVC salvage may sometimes outweigh the risk of a delayed removal. In such cases, the so-called “lock therapy” (LT) has been suggested as a possible therapeutic strategy (81, 82), although, there is still insufficient experience concerning its use in neonates, and its actual efficacy and safety are still discussed by authors. The LT consists of high concentrations of antibiotic drugs instilled into the lumen of the catheter and left *in situ* for few hours. LT with antifungal drugs has been suggested as a possible therapeutic option for central venous catheter (CVC)-related fungal infections, whenever critical clinical conditions of a neonate make it difficult, or dangerous to remove the catheter (82). In neonates, infections due to the use of CVC are mostly related to the development of biofilms inside the catheter surface (83). Biofilms are made up of microbial cells embedded in a self-secreted polymeric matrix (made of water, polysaccharides, proteins, lipids, and extracellular DNA) released in the extracellular space (84). This matrix provides a protective barrier able to decrease the penetration of antimicrobials and to provide the fungal colonies protection against mechanisms of host immune defense (10). Therefore, biofilms may become a reservoir for systemic spread to other sites of the body.

The optimal antimicrobial drug to be used for a LT should be identified according to the specific antibiogram. Very high concentrations of antimicrobial drugs, 100 to 1,000 times the microorganisms' MIC, should be instilled into CVC lumen for a determined dwell-time. If the antibiogram is unavailable, a LT with ethanol 70% could be performed. In case of *Candida* infection, micafungin should be preferred to other antifungal drugs such as amphotericin B or fluconazole, thanks to its ability to penetrate the biofilm (63). However, a recent Cochrane review concluded that, although preventive LT appeared to be effective in decreasing the incidence of catheter-related bloodstream infections, the evidence was still insufficient to recommend it, considering the limited number of trials and the heterogeneity of antimicrobial drugs administered and about the optimal LT dosage and timing (85). Piersigilli et al. described the case of a preterm infant with critical CVC-related *Candida albicans* infection unresponsive to the systemic therapy. The infant received a combined LT [1:1 mixture of 70% ethanol and micafungin sodium 5 mg/L], which allowed for the resolution of the infection and the preservation of his long-term CVC (67). However, further studies are required to confirm the efficacy and safety of the LT in neonates and to design the most appropriate dosage and dwell times.

### Therapeutic Strategies for the Treatment of Fungal Abscesses

Hepatic fungal abscesses are uncommon in neonates. They usually occur in the course of fungal sepsis, due to the localization of the germ within the hepatic parenchyma. *Candida albicans* is the most common fungal organism isolated (74). Cases of hepatic fungal abscesses described in the literature are sporadic (75, 76, 86–88) and they mainly involve preterm infants due to the functional immaturity of the immune system and the invasive procedures necessary for preterm babies to survive. Among the recognized risk factors, the strongest is the presence of vascular catheters, especially umbilical catheters. Other important risk factors are the presence of bowel diseases requiring surgery, such as isolated intestinal perforation, necrotizing enterocolitis, and the concomitant presence of sepsis (74). Diagnosis of fungal abscesses is challenging. The optimal therapeutic approach is still uncertain and mortality is high, due to the ineffectiveness of medical therapy alone. In adults, hepatic abscesses are treated by percutaneous needle aspiration or by percutaneous catheter drainage, associated with medical therapy. Patients who fail to respond to such a treatment undergo surgery. In preterm infants, especially those with a very low birth weight, the extreme difficulty in performing needle aspirations should be considered (89). Abscess culture is recommended to assess microbiological sensitivity pattern of the organism, and start the most appropriate therapy. Auriti et al. reported the case of a *Candida albicans* hepatic abscess in a severely ill preterm neonate, successfully treated by intralesional administration of L-AMPH-B (1 mg/ml, in isotonic water) (90). Further investigations are required to confirm such practice.

### Shunt Lock Therapy With Antifungal Drugs in Neonates With Hydrocephalus

In case of hydrocephalus, potential complications such as shunt-associated fungal infections may occur, representing a challenging situation of the early life periods. Such infections are currently treated with systemic antifungal drugs. As already mentioned above in the text, recent studies measuring micafungin concentrations in CSF demonstrated that this drug, particularly in patients treated with high doses, has enough penetration in the CNS to have a good antifungal effect (30, 68, 73). International guidelines, however, recommended shunt replacement, whenever possible, because of the ability of fungi to form, inside the lumen, a biofilm resistant to antifungal therapy, with lasting CSF positive cultures despite optimal therapy (91). Considering the ability of echinocandins to eliminate fungal biofilms from CVCs (92), Auriti et al. successfully treated a shunt-associated *Candida albicans* meningitis by means of systemic antifungal therapy combined with micafungin LT of the external ventricular drain (EVD) in a seriously ill, preterm infant with posthaemorrhagic hydrocephalus (68). The use of shunt LT needs a larger validation to determine the optimal duration and number of locks to sterilize the shunt or to prevent recolonization after new EVD insertion.

## Conclusions

Fungal infections represent severe complications of the neonatal period, associated with high morbidity, and mortality. Diagnosis is challenging and requires long-lasting diagnostic testing such as cultures. As such infections are difficult to eradicate by means of the traditional treatments, specific therapeutic strategies developed in the last years, such as catheter LT and shunt LT in association with the systemic therapy may provide additional efficacy of antifungal treatments. Further investigations are required to confirm this hypothesis.

## Author Contributions

All authors listed have made a substantial, direct and intellectual contribution to the work, and approved it for publication.

### Conflict of Interest

The authors declare that the research was conducted in the absence of any commercial or financial relationships that could be construed as a potential conflict of interest.
